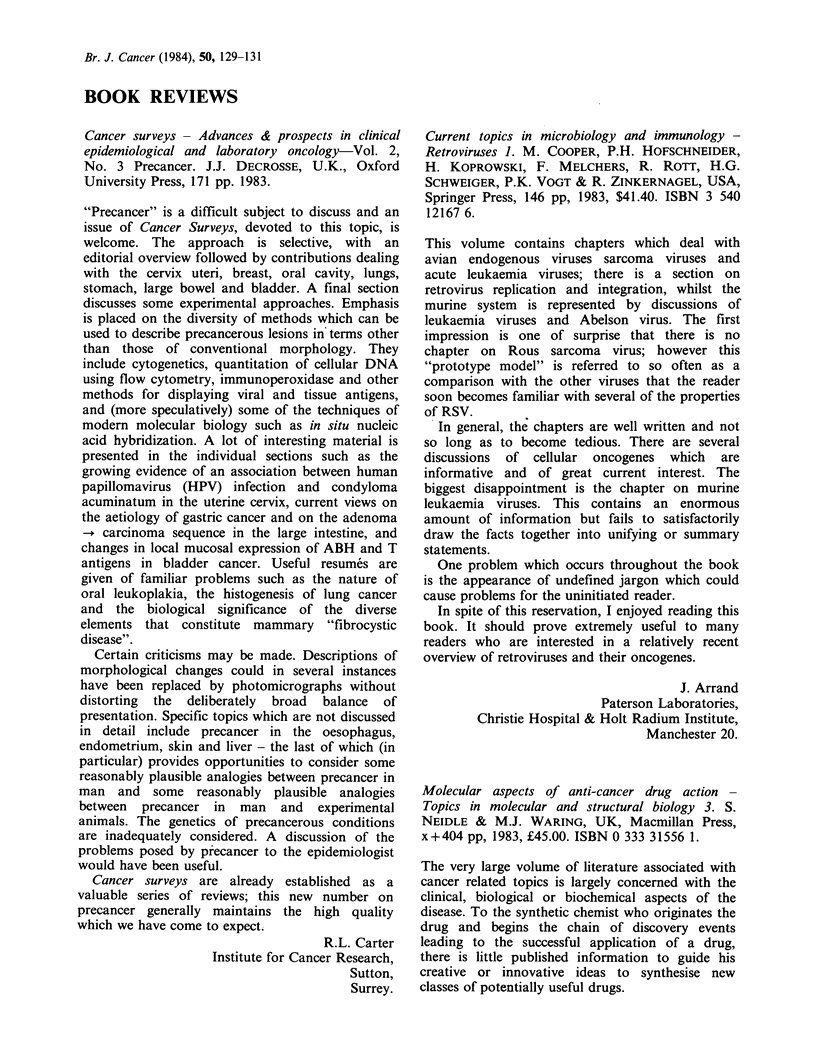# Current topics in microbiology and immunology - Retroviruses

**Published:** 1984-07

**Authors:** J. Arrand


*Current topics in microbiology and immunology – Retroviruses 1.* M. COOPER, P.H. HOFSCHNEIDER, H. KOPROWSKI, F. MELCHERS, R. ROTT, H.G. SCHWEIGER, P.K. VOGT & R. ZINKERNAGEL, USA, Springer Press, 146 pp, 1983, $41.40. ISBN 3 540 12167 6.

This volume contains chapters which deal with avian endogenous viruses sarcoma viruses and acute leukaemia viruses; there is a section on retrovirus replication and integration, whilst the murine system is represented by discussions of leukaemia viruses and Abelson virus. The first impression is one of surprise that there is no chapter on Rous sarcoma virus; however this "prototype model" is referred to so often as a comparison with the other viruses that the reader soon becomes familiar with several of the properties of RSV.

In general, the chapters are well written and not so long as to become tedious. There are several discussions of cellular oncogenes which are informative and of great current interest. The biggest disappointment is the chapter on murine leukaemia viruses. This contains an enormous amount of information but fails to satisfactorily draw the facts together into unifying or summary statements.

One problem which occurs throughout the book is the appearance of undefined jargon which could cause problems for the uninitiated reader.

In spite of this reservation, I enjoyed reading this book. It should prove extremely useful to many readers who are interested in a relatively recent overview of retroviruses and their oncogenes.

J. Arrand
Paterson Laboratories,
Christie Hospital & Holt Radium Institute,
Manchester 20.